# Ulcerated Legs : Their Care and Cure

**Published:** 1887-02-05

**Authors:** 

**Affiliations:** Founder of St. Mary's Cottage Hospital, Southampton


### Ulcerated Legs; Their Care and Cure.

By Mrs. Black,
Founder of St. Mary's Cottage Hospital, Southampton.

More than fourteen years ago, whilst district visiting, I came upon my first experience of the "Ulcerated leg," or, as it is commonly called by the poor sufferers themselves, a "bad leg." The patient was a sober, respectable mason, aged forty-five, and the malady had lasted twenty-five years, during which time he had constantly been an in-patient at hospitals. At last the ulcer increased so rapidly, he was obliged to give up his work. I collected a small supply of money to maintain him, and his family (twelve in number) for four months, during which time I visited and dressed the sore twice daily. It healed completely, and to this day remains sound. He has had constant work, and periodically reports himself at the hospital. Many similar sufferers applied to me, and as it was impossible to go to all, I hired a room at sixpence per hour, where I went every morning; and in a few days I had twenty cases. I then wrote a letter to all the local papers, which was promptly inserted by my courteous and kind friends—the Editors--briefly describing the nature of the work, and appealing to the charity of the public—for funds to continue. It was instantly and generously responded to. The landlady of the humble apartment assisted me in dressing and bandaging the sores, and has been for fourteen years the faithful, conscientious and invaluable matron of the St. Mary's Cottage Hospital, Southampton, the only institution in the country which is exclusively devoted to the care and cure of ulcerated legs. The young daughter of the matron—then little more than a child—worked with us, and has been for many years the head nurse. The ever increasing numbers soon outgrew the little room, and funds coming in, justified the renting of a tiny house where the matron lived, and where cases were treated daily as out-patients. This house was formally opened by the Rev. Canon Wilberforce, on the 10th of May 1874.

By this time many kind friends had become annual subscribers. Donations had flowed in, and the funds increased by sundry entertainments, concerts, etc., which were always well supported. A report was issued annually. At the end of the third year, a committee was formed, trustees appointed, and honorary auditors. Junior nurses were engaged for three hours twice a week and were trained accordingly. In the second year of work, the hospital became a participator in the local "Sunday Fund," and its income was further augmented some years later by a concert given (by kind permission) at Dudley House, and a ball at the Kensington Town Hall. In 1880 a house was purchased, which is now the permanent Cottage Hospital. It is opened every morning from 10 to 1, except on Saturdays, when the hours are from 3 to 5. All the ointments, washes, and lotions, are made on the premises by the matron, and are entirely under her management. We have seldom been without the kind and gratuitous services of some able physician or surgeon who would prescribe and even make up the medicines, which were kept in stock, but the immense numbers who flocked to it from all parts necessitated the fixing up of a small dispensary, and the services of a paid dispenser, for two hours every Wednesday morning. Our kind Honorary Surgeon, F. Hall Esq., attends punctually once a week to prescribe for such cases as need medicine.

In some cases where the poverty of the patient is very apparent, tickets for grocery and meat are issued, but it is obvious this can only be done with a sparing hand. The patients come from long distances with most commendable regularity, few needing to be admonished to come more frequently. Those who can afford it, pay in part for their bandages, and notices are fixed on the walls, requesting all who are able, to drop their pence in the box. It was originally intended to have one or two beds, but independent of the inadequacy of the funds, the difficulty of working this, without a more extensive staff, and the disproportion of the benefits derived, with the expenses it would entail, and a belief, favoured more and more by experience that very few cases would arise requiring such treatment, led to the idea being abandoned.

The fourteenth and last Report issued in November 1886, shows that:—

| | |
|---|---|
| The number of attendances during the year for dressing sores has amounted to - - - - - - | 7,011 |
| The number of attendances on the Doctor for medicines supplied by the Hospital - - - - - - | 2,054 |
| The number of dressings sent by post or carrier to patients, who, from great distances are only able to attend occasionally - - - - - - | 854 |
| The number of visits paid by Matron or Head Nurse to such patients at their own dwellings, who are only able to attend occasionally - - - - - | 696 |

The expenditure for the year has been £261 12*s*. 8½*d*.

[Few general readers can realise the amount of misery, disappointment, and destitution which arises amongst the poorer classes from neglect of so-called "bad legs" and the want of provision for their prolonged and successful treatment. The above 10,000 attendances and dressings represent the restoration of many a breadwinner to his family, and all who feel interested in this most useful work are invited to write direct to Mrs. Black, St. Mary's Cottage Hospital, Southampton. —Ed. T. H.]

